# ﻿A reappraisal of Thunberg’s spotted lily *Lilium maculatum* Thunb. and recognition of the elegant lily Lilium × elegans Thunb. (Liliaceae)

**DOI:** 10.3897/phytokeys.263.163611

**Published:** 2025-10-09

**Authors:** James A. Compton

**Affiliations:** 1 Spilsbury Farm, Salisbury, SP36RU, UK Spilsbury Farm Salisbury United Kingdom

**Keywords:** Dejima, Japan, *Lilium* × *elegans*, *L. maculatum*, nomenclature, Thunberg

## Abstract

Thunberg encountered nine species of *Lilium* during his visit to Japan in 1776. Of these, *L. lancifolium* Thunb., *L. longiflorum* Thunb., *L. maculatum* Thunb., and *L. speciosum* Thunb. are all accepted. He also described *L. elegans* Thunb., a lily that is morphologically similar to *L. maculatum*. The long-disputed name L. × elegans is confirmed as correctly belonging to the hybrid comprising *L. maculatum* and *L. pensylvanicum* Ker Gawl. Thunberg’s working methodology, including that of his illustrators and engravers, is scrutinised concerning the publication of his Japanese lilies. Four lectotypes and a neotype are designated, a full synonymy is listed for *L. maculatum* and L. × elegans, and keys to the hybrid and its parent species, as well as to the natural varieties within *L. maculatum*, are provided.

## ﻿Introduction

Sixteen recognised species of the genus *Lilium*L. occur in Japan (Hayashi 2016: 111). Two of these, *Lilium maculatum* Thunb. and *L. pensylvanicum* Ker Gawl., have scattered leaves and upright, bowl- or cup-shaped floral morphology with tepals that narrow to a claw-like base. *Lilium maculatum* is endemic to Japan, while *L. pensylvanicum* has a much wider distribution across much of temperate to sub-arctic eastern Asia (Compton and Sytin 2023). Recent molecular data have shown that their nearest relative is the European species *L. bulbiferum*L. (Du et al. 2014: 254; Huang et al. 2018: 1253; Givnish et al. 2020: 38; Watanabe et al. 2021: 193). These data have also shown that the two Asian species and the single European species belong together with seven Asian species in *L. sect. Sinomartagon* H.F.Comber, and have also been placed together in *L. subsect. Dahurica* Baranova, classifications based originally on morphology (Comber 1949: 101; Baranova 1988: 1327). *Lilium sect. Sinomartagon* is now characterised as having adventitious stem roots above the bulb; stems more or less striated; a marginal ridge on either side of a sinus on the adaxial surface of the tepals; and short papillae present on the stigmas (Watanabe et al. 2021: 199, 201).

Two North American species, *L. catesbaei* Walter and *L. philadelphicum*L., share several similar floral morphologies with the Eurasian species, such characters leading botanists in the past to classify them together under what John Gilbert Baker referred to as Group 3 “Isolirion” (Baker 1871: 104; Wallace 1873: 63). According to molecular data (Du et al. 2014: 254; Huang et al. 2018: 1253; Givnish et al. 2020: 38; Watanabe et al. 2021: 193), these two North American species are not closely related to the Eurasian species and belong together in a different section of the genus, *Lilium sect. Pseudolirium* (Endl.) Spach (Spach 1846: 277). Close examination of these North American species reveals that they can also be distinguished from the Eurasian species by their glabrous stems, leaves, and flower buds, and by their tepals, which narrow abruptly towards the base, forming a linear furrow.

*Lilium bulbiferum*, the only European species in *L. sect. Sinomartagon*, is characterised by having small bulbils produced in the leaf axils along the inflorescence axis, although bulbils may be lacking in var. croceum (Chaix) Pers., but with terete stems with less pubescence on the inflorescence axis than that on its Asian counterparts. This species, typified on a specimen clearly having bulbils LINN 420.2 (Peruzzi and Jarvis 2009: 1361), is undoubtedly closely related to both *L. maculatum* and *L. pensylvanicum*, sharing similar morphologies and molecular data (Du et al. 2014: 254; Huang et al. 2018: 1253; Givnish et al. 2020: 38; Watanabe et al. 2021: 193). It is, however, restricted to Europe and is not the focus of this paper.

There is little doubt that *L. maculatum* and *L. pensylvanicum* closely resemble each other morphologically. Their geographical distributional ranges only overlap in the most northern extremity of Honshu Island, where *L. pensylvanicum* has its most southerly populations in Japan (Hayashi 2016: 111, 112; Watanabe et al. 2024: 467). *Lilium pensylvanicum* has ribbed stems with floccose pubescence distributed on the upper stem parts, particularly in the leaf axils and along the flower buds and pedicels, and has hypogeal seed germination. *Lilium maculatum* has scarious stems with papillose hairs and epigeal seed germination (Hayashi 1990: 9; 2016: 112; Watanabe et al. 2024: 460). *Lilium pensylvanicum* occurs throughout much of central and eastern Siberia, the Korean Peninsula, Manchurian China, and Mongolia. It also occurs in Sakhalin and the southern Kuril Islands (Compton and Sytin 2023). In Japan, it is found across Hokkaido and in a few places in the extreme north of Honshu, on Shimokita Peninsula and around Cape Tappi on the Tsugaru Peninsula, both in Aomori Prefecture (Hayashi 2016: 112).

A recent conservation proposal by Y-D. Gao to conserve the name *L. dauricum* Ker Gawl., a name widely used in the past, over *L. pensylvanicum* (Gao 2021: 1139–1140), has been rejected (Applequist 2023: 917–918). This conclusion means that *L. pensylvanicum* remains the correct name for that east Asian species. The full range of names associated with *L. maculatum* is discussed below (see synonymy).

The east Asian morning star lily, *L. concolor* Salisb., which also belongs in *L. sect. Sinomartagon*, was so named for its flowers with much narrower tepals which radiate in a stellate manner (Hayashi 2016: 112). An article by Fritz Berckmüller of Hamburg indicated that Thunberg’s *L. maculatum* (under the name *L. thunbergianum*) was of hybrid origin with *L. pensylvanicum* (as *L. dauricum*) and *L. concolor* as the parent species (Berckmüller 1927: 216). This hybrid identity was then taken up under the name L. × maculatum by later workers (e.g. Stearn 1948: 12; Woodcock and Stearn 1950: 263; Synge 1980: 64). The wide range of variation exhibited in horticultural selections of plants previously identified as *L. maculatum* is likely to have enhanced that conclusion. Characters such as robustness of plant stature, increased number of flowers, variable tepal colouring, and ornamentation on the tepals are strong indicators of potential hybridity. A very similar lily to both *L. maculatum* and *L. pensylvanicum*, described by Thunberg as *L. elegans* Thunb. (Thunberg 1811: 203), has also been postulated to be of hybrid origin with *L. pensylvanicum* and *L. maculatum* as the putative parent species (e.g. Shimizu 1960: 105; 1969: 27; 1973: 119; Furuya 2009: 85; Okubo 2014: 12). Recent research based on morphology as well as DNA sequence data from plastid and nuclear genes and gene spacer regions has confirmed the existence of this hybrid, although no hybrid formula was provided in that work (Watanabe et al. 2024: 457, 467). In this paper, the hybrid status of both L. × elegans and L. × maculatum is discussed. A critical overview of Thunberg’s working methods, a review of his publications, and a thorough examination of his herbarium specimens with respect to Japanese *Lilium* names has been undertaken. An appraisal is made of all the subsequent names associated with these lilies, and their statuses are assessed.

## ﻿Thunberg’s discovery of the spotted lily

*Lilium maculatum* Thunb. was first described by the Swede Carl Peter Thunberg (1743–1828) in the second volume of the British journal Transactions of the Linnean Society of London (Thunberg 1794: 334). His description of the species was made within the commentary on his major work, Flora Japonica, published a decade earlier (Thunberg 1784). Thunberg, who was one of Linnaeus’s students, collected material of the lily in the summer of 1776 during the 15 months which he spent in Japan. It was not possible at that time for “foreigners” to travel anywhere in Japan. The Chinese were permitted a small enclave outside the city of Nagasaki, and the Dutch were restricted nearby to the small artificial island of Dejima in Nagasaki Bay. This island, which was to all intents and purposes a tiny Dutch colony, was linked by a guarded bridge to the large southern Japanese island of Kyushu. The Dutch were the only European nation permitted by the Japanese to trade with them, but only from this small island. Thunberg’s travels while he was in Japan, always under a Japanese escort, were consequently restricted to the locality around Dejima, the exception for him being the annual *hofreis* or court journey, an expedition undertaken by the Dutch in order to pay homage to Japan’s military ruler, the shōgun, in Edo [Tōkyō] on Honshu. This rule of restricted movement came under the *sakoku* or “locked in” policy, which excluded all foreigners from setting foot on Japanese soil without permission and was not revoked until almost 100 years after Thunberg’s time there.

Although he was Swedish and had several eminent Swedish sponsors, including Linnaeus, his journey to Japan was sanctioned and sponsored on behalf of the Dutch East India Company (VOC) by several influential Amsterdam burghers, including his contemporary the alderman Jean [Johann] Deutz (1743–1788), a director of the Surinam Society and correspondent of Joseph Banks; burgemeester Egbert de Vrij Temminck (1700–1785); and the city councillor, director of the Surinam Society, and burgemeester Jan van de Poll Pietersz (1726–1781). Also much involved in promoting Thunberg’s employment as a physician for the VOC was Linnaeus’s friend and colleague the Dutch botanist Johannes Burman (1707–1780), professor at the Amsterdam Athenaeum Illustre [later University of Amsterdam] (Skuncke 2014: 58). Thunberg was commissioned initially to visit the Cape in South Africa, where, from 1772 to 1775, he learnt Dutch. His final destination, however, was Japan, where he would be based as the physician in the Dutch *factorij* or trading post on Dejima, which Thunberg soon found out was to become a virtual prison.

The Japanese name of *Lilium maculatum* is *sukashi yuri* スカシユリ, a phrase meaning “see-through lily”, a reference to the open spaces between the bases of each tepal. The species is restricted to Honshu, growing on sandy banks and rocky ledges from Tōhoku in the north of the island southwards to the central Chūbu region (Hayashi 2016: 112). Thunberg never visited the Tōhoku region as it was far too distant from the places he was permitted to travel in the southern parts of Japan. He did, however, pass through eastern coastal parts of the Chūbu region in Honshu (see below).

The annual *hofreis* or court journey must have been a great escape from Thunberg’s confinement. Each year, the Dutch on Dejima had to pay homage to the de facto ruler of Japan, the shōgun Tokugawa Ieharu, in faraway Edo on Honshu. This homage involved a land and sea journey covering a distance of 2,600 km, which, including the return journey, lasted for several months. On 4 March 1776, the Dutch chief merchant on Dejima, Arend Willem Feith, accompanied by Thunberg, as physician for the Dutch, and the scribe Herman Köhler, set out on their journey. Their route took them along the Pacific coast of the Chūbu region of Honshu, through parts of modern-day Shizuoka Prefecture where the lily still grows (Kijewski 2015: 162, map fig. 2).

Thunberg recounts his journey in the Preface to Flora Japonica (Thunberg 1784: xviii):

“*Annus 1776 mihi et Florae dilectissimae maxime favebat; iter enim ad aulam sero susceptum, solito diutius protrahebatur, ob adversos in mari ventos 130 milliaribus propius ad littora confectis. Hinc ver praeteribat, aestas vero adventabat, ut in reditu plurimas inuenerim plantas eximie florentes.Dum in Metropoli Iedo versabar, ab 18 Maii ad 13 Jun. in diversorio saepissime me adibant duo medici Kafragawa Fosiu et Nakagawa Sunnan, in Mineralogia, Zoologia, Botanica et Medicina haud parum versati* [The year 1776 was most favourable to me and to my beloved Flora; for the court journey, having been undertaken late, extended longer than usual, owing to adverse winds at sea, we found ourselves for 130 miles closer to the coast. From here, the spring passed away, and the summer came on, so that on my return, I found many plants blooming exquisitely. While I was in the metropolis of Yedo (Tokyo), from the 18^th^ of May to the 13^th^ of June, two doctors, Kafragawa Fosiu and Nakagawa Sunnan, who were well versed in mineralogy, zoology, botany, and medicine, visited me very often in my dwelling] (Thunberg 1784: xviii).

His translation of the Japanese names Kafragawa Fosiu and Nakagawa Sunnan was probably intended to refer to the physician and translator Katsuragawa Hoshū (1751–1809) and the surgeon Nakagawa Jun’an (1739–1786). As well as wild plants in Shizuoka, it is also likely that Thunberg saw flowering plants of *L. maculatum* and *L. elegans* cultivated in the gardens wherever he had stopped along the route during his return journey from Edo via Miako [Kyōto] and Ōsaka on Honshu on his way back to Dejima, Nagasaki, on Kyushu.

## ﻿Thunberg’s Flora Japonica

Thunberg’s first contribution to the classification of the lilies of Japan was in his Flora Japonica (Thunberg 1784: 133–135). Of the eight lilies he had included (Table 1), only one species, *L. japonicum* Thunb. ex Houtt., still has the correct name (Thunberg 1784: 133). It was described in 1780 by the Dutch botanist Maarten Houttuyn from one of Thunberg’s collections and published four years before Thunberg’s own account (Houttuyn 1780: 245). Prior to his arrival in Japan, Thunberg’s knowledge of the genus *Lilium* in that country was inevitably limited to accounts that had been previously provided by a handful of Europeans who had been able to gain access to some knowledge about Japanese plants. In this context, three of Thunberg’s species names are important in helping to understand his concept of what these Japanese species may have represented to him: the European *L. bulbiferum*L. (Linnaeus 1753: 302) and two North American species, *L. canadense*L. (Linnaeus 1753: 303) and *L. philadelphicum*L. (Linnaeus 1762: 435). His initial species determinations in Japan, therefore, were influenced by his understanding of species which had previously been described from elsewhere in the world. Subsequent examination of his herbarium specimens, carefully scrutinised along with the annotations on his sheets, has proved pivotal in correctly determining the identity of several of his taxa.

**Table 1.** Chronology of Thunberg’s determination and naming of Japanese *Lilium* species.

Flora Japonica 1784	Observations 1794	Examen 1811	Currently accepted name and specimen in UPS
*L. bulbiferum*	*L. lancifolium*	*L. lancifolium*	*L. lancifolium* V-008139
*L. canadense*	–	–	L. leichtlinii subsp. maximowiczii
*L. candidum*	*L. longiflorum*	*L. longiflorum*	*L. longiflorum* V-008140
*L. japonicum* var. α	–	*L. japonicum*	*L. japonicum* V-008137
*L. japonicum* var. β	–	–	*L. japonicum*
*L. philadelphicum*	*L. bulbiferum*	*L. elegans*	L. × elegans V-008129
*L. pomponicum*	–	*L. pomponicum*	*L. concolor* V-008147
*L. superbum*	*L. speciosum*	*L. speciosum*	*L. speciosum* V-008149
–	*L. cordifolium*	*L. cordifolium*	*Cardiocrinum cordatum* V-008135
–	*L. maculatum*	*L. maculatum*	*L. maculatum* V-008141

When considering Thunberg’s determinations, it should also be borne in mind that after he had left Japan on 3 December 1776, he did not return to his native Sweden for over 3 years, having spent time travelling and collecting in Java, Sri Lanka, the Cape of Good Hope, and Sierra Leone. Moreover, he also spent 2 months studying in Amsterdam and London, finally reaching Ystad in southern Sweden on 14 March 1779 (Skuncke 2014: 186). After his arrival in Sweden, it was another 5 years before his Flora Japonica was eventually published in the Upper Saxon city of Leipzig (Thunberg 1784). Over that period, some of his notes were likely to have become confused, and his initial recollections of the taxa concerned may have become less than accurate. The late expert on *Lilium*, Professor William Thomas Stearn (1911–2001), writing about *L. japonicum* and specifically about Thunberg’s compilation of Flora Japonica, made the following apposite comment: “His Flora Japonica wherein no less than eight lilies were recorded from Japan, is full of erroneous determinations; it served nevertheless as a basis for later work…” (Stearn 1947: 180).

In the Preface to his Flora Japonica, Thunberg cited works connected to Japan and, in particular, to three earlier European botanist-physicians (Thunberg 1784: xxvi). He mentioned Christian Mentzel (1622–1701), who, as personal physician to Friedrich Wilhelm, Elector of Brandenburg, would have seen the *Honzō kōmoku* translated and published in Kyōto by Noda Yajiemon in 1637 and which was kept in the elector’s Berlin library. This was the first Japanese printing of the Chinese *Ben Cao Gang Mu* [Compendium on Materia Medica], compiled by the Chinese 16^th^-century herbalist Li Shizhen (1518–1593), comprising woodcuts of plants, including species of *Lilium*, minerals, and animals, originally published posthumously in 1596 in Nanjing, China.

Mentzel also received the 599 coloured drawings of Japanese plants which were sent back to him by the German Andreas Cleyer (1634–1698), the *opperhoofd* or chief merchant on Dejima (Michel 2002: 715). Cleyer and his assistant and gardener Georg Meister (1653–1713) were posted on Dejima twice, firstly from 1683 to 1684 and again from 1685 to 1686. Mentzel had the illustrations bound into one volume with a frontispiece titled Flora Japanica (Mentzel 1695), which is now in the Staatsbibliothek in Berlin (shelf mark *Libri picturati* A 41/42). Cleyer wrote two short essays on Japanese lilies (for more details, see Lack 2014). The first, entitled *Observatio CXCI De Floribus Japanensibus, Kanako Juri et Jama Juri* (Cleyer 1690: 490), includes two figures: Cleyer 1690: fig. 53, with stem bulbils in the leaf axils and spotted reflexed flowers, titled *Jama Juri* [mountain lily], which represents *L. lancifolium* Thunb.; and Cleyer 1690: fig. 54, titled *Kanoko Juri* [desirable lily], which is readily identifiable as *L. speciosum* Thunb. (Cleyer 1690: 490). The second is more or less a repeat of the first, entitled *Observatio LXXVI Floribus Japanensibus Vohsnofana & Ghimi* (Cleyer 1691: 127), accompanied by Cleyer 1691: fig. 20 “Ghimi sive Musme Juri”. His description of it having edible leaves and bulbs and brick-coloured flowers with a reflexed perigone makes it identifiable as L. leichtlinii Hook.f. subsp. maximowiczii (Regel) J.Compton. Meister also wrote about lilies in his treatment of Japanese useful plants in his work *Der Orientalisch-Indianische Kunst-und Lust-Gärtner* [The Oriental-Indian art and pleasure gardener], in which he referred to what he called “Schrogury Lilium Album” (i.e. *shiro juri*, meaning white lily), which was probably *L. longiflorum* Thunb., and “Cannakogury” (Cleyer’s *kanoko juri*) or *L. speciosum* (Meister 1692: 163).

In the same passage of his Preface in Flora Japonica, Thunberg mentioned Christian Heinrich Erndel (1676–1734), botanist-physician to Friedrich Augustus, Elector of Saxony (also known as Augustus II the Strong), who published texts in 1716 on Japanese medicinal plants compiled by the Danzig [Gdańsk] merchants Jacob Breyne (1637–1697) and his son Johann Philipp Breyne (1680–1764). As wealthy merchants, the Breynes’ publication was based on the Japanese collections of the VOC and, in particular, on those collections and publications of Cleyer and Meister already mentioned.

Thunberg, however, singled out the work of his predecessor on Dejima, the German physician Engelbert Kaempfer (1651–1716), during the latter’s visit as physician for the VOC on the little island colony from 1690 to 1692. Kaempfer made the *hofreis* to Edo on two occasions in 1691 and 1692 and later published his botanical research in *Amoenitatum exoticarum* (Kaempfer 1712). Thunberg also attempted to learn the Japanese names of the plants which Kaempfer had included.

Kaempfer included eight plants (Kaempfer 1712: 870–872) which he categorised as lilies: 1. Sjirè, rectius Sjirói & Osjiroi Lilium album, which he called white lily, and judging from his description of “Calthae palustris folio, caule sesquicubitali pingui” meaning leaves like *Caltha palustris*L. stem thick, 27 inches = c. 68 cm tall, possibly referred to the related lily-like *Cardiocrinum cordatum* (Thunb.) Makino; 2. Jamma Osjiroi, which the description states is a woodland plant with three leaves clasping the stem, could be *Trillium tschonoskii* Maxim.; 3. Biakko, vulgo Juri, with scented flowers, which may refer to Polygonatum odoratum (Mill.) Druce var. thunbergii (C.Morren & Decne.) H.Hara; 4. Sazuri probably *Lilium longiflorum*; 5. Kentan, vulgo Oni Juri, Lilium diaboli, known in Japan as the devil’s lily *Lilium lancifolium*; 6. Kasbiako, vulgo Kónnoko Juri identifiable as *L. speciosum*; 7. Santan, vulgo Fime Juri, which, from the description of flowers like a small *Fritillaria imperialis*L. but purple-blood red and spotted, might refer to *Fritillaria camschatkensis* (L.) Ker Gawl. and 8. Fi Juri Lilium igneum, meaning fire lily, might refer to *L. callosum* Siebold & Zucc.

When Thunberg arrived in Japan, encountering hitherto unknown Japanese species of *Lilium*, these references, with their frequently rather inaccurate depictions and descriptions, were his primary sources for consultation.

## ﻿Thunberg’s first concept of *Lilium* for Flora Japonica

Thunberg included eight lilies in his *Flora Japonica* (Table 1), which he called: *L. bulbiferum*, *L. canadense*, *L. candidum*, *L. japonicum*, *L. philadelphicum*, *L. pomponicum* [sic!], and *L. superbum*, as well as two varieties of his new species *L. japonicum*, vars. α and β (Thunberg 1784: 133–135). He clearly believed that Japanese plants representing *L. bulbiferum* and *L. candidum*L. were the same as their European counterparts, citing Linnaeus’s descriptions for those species verbatim (Linnaeus 1753: 302), including references to Linnaeus’s species names in each case. *Lilium bulbiferum* is confirmed as having axillary bulbils along its stem (Peruzzi and Jarvis 2009), which undoubtedly led Thunberg to conclude erroneously that he was observing the same species.

Thunberg also included under the name *L. “pomponicum*” the plant with red Turk’s cap flowers that he believed to represent the European *L. pomponium*L. (Linnaeus 1753: 303), and another as the North American *L. superbum*L. (Linnaeus 1762: 434). As far as Thunberg’s concept of *L. bulbiferum* was concerned, he recognised that the Japanese lily had scattered leaves and a campanulate upright corolla, but did not elucidate further. He cited Kaempfer’s description of the species (Kaempfer 1712: 871) and repeated Kaempfer’s Japanese names for it, “Kentan”, “Oni Juri”, and “Jamma Juri” [mountain lily], but excluded Kaempfer’s citation of “*Lilium diaboli*” or devil’s lily (Thunberg 1784: 134). There is a specimen showing bulbils in the leaf axils up the stem but lacking flowers in Thunberg’s herbarium in Uppsala [UPS-THUNB 8139] with an annotation written in Thunberg’s hand “*Lilium lancifolium*” and on the reverse “e Japonia Thunberg”. This is clearly identifiable as *L. lancifolium*, not *L. maculatum*.

Thunberg, however, also included the same Japanese names “Kentan”, “Oni Juri”, and “Jamma Juri” for the species, which he called *L. canadense* on the following page (Thunberg 1784: 135). Thunberg again repeated Linnaeus’s phrase name for *L. canadense* verbatim and stated that this lily had leaves in whorls and reflexed flowers, but also noted “hinc inde in sylvulis, Nagasaki montibus, alibi” [found here and there in woods, in the mountains around Nagasaki, and elsewhere] (Thunberg 1784: 135). In Thunberg’s herbarium in Uppsala, there is a cultivated specimen that was annotated “*Lilium canadense*” and on the reverse “cult. in Horto Uppsaliensis. Thunberg” in Thunberg’s hand [UPS-THUNB 8130]. This specimen has scattered leaves and flowers with narrow, recurved tepals covered with many spots and is referable today to L. leichtlinii subsp. maximowiczii (Regel) J.Compton (Compton 2021). This discrepancy over names serves to highlight Thunberg’s initial rather contradictory determinations of Japanese lilies.

Thunberg added further grist to the mill of confusion by including what he believed to be another North American lily species, *L. philadelphicum*, which had also been described by Linnaeus (Linnaeus 1762: 435). Thunberg once again repeated Linnaeus’s descriptive phrase name for that species verbatim, describing this lily as having whorled leaves, upright flowers, and petals with claws (Thunberg 1784: 135). There is no specimen in Thunberg’s herbarium with this name attached.

## ﻿Thunberg’s second concept of *Lilium* in Observations on the Flora Japonica

Another decade passed before Thunberg published the Botanical Observations on the Flora Japonica, which was a commentary with revisions on the species he had outlined in his earlier work. The Observations were read out to the Linnean Society of London on 1 October 1793 (Thunberg 1794: 332–334). With respect to *Lilium*, Thunberg discussed six species (Table 1), one of which, *Lilium cordifolium* Thunb., is now recognised as belonging in a related genus as *Cardiocrinum cordatum*, based on his earlier *Hemerocallis cordata* Thunb. In the Observations, four new species of *Lilium* were described: *L. lancifolium*, *L. longiflorum*, *L. maculatum* and *L. speciosum*. He also included, as the remaining species of the six in the work, his second concept of *L. bulbiferum*, citing as a synonym, the *L. philadelphicum*, which he had previously cited in Flora Japonica. This was a clear indication that he had changed his mind about the identity of one of the plants, which he had originally called *L. bulbiferum* in Flora Japonica, referring to that instead as a synonym of his new species *L. lancifolium* (Thunberg 1794: 333). Thunberg’s decision was influenced by Kaempfer’s description “gemmis in sinu foliorum pisi magnitudinis” and “flores …. sericeis dense guttato” [bulbils in the pockets of the leaves which are the size of peas] and [flowers ….. densely clothed in silky hairs]. Thunberg realised that this description applied equally well to the European *L. bulbiferum* as to his new Japanese species, which also produced small bulbils along the stem in the leaf axils. The tiger lily, *L. lancifolium*, known for some time under the synonym *L. tigrinum* Ker Gawl., is now typified on Thunberg’s sheet UPS-THUNB 8139 (Gao and Gao 2017: 174). It is found throughout all four major islands of Japan, is widely cultivated, and its diploid form is only found on the island of Tsushima, which lies between Kyushu and South Korea (Hayashi 2016: 114).

In the protologue for *L. maculatum*, Thunberg provided the Latin diagnosis: “foliis sparsis et verticillatis lanceolatis, glabris, corollis campanulatis intus maculatis: limbo reflexo.” [leaves sparse, verticillate, lanceolate, glabrous, corollas campanulate, spotted within: limb reflexed]. He described the leaves as sparse and whorled, sessile, lanceolate, glabrous, multinerved and finger-like; the corollas campanulate with the limb reflexed, flesh-pink and inside they were liberally spotted with many purple spots (Thunberg 1794: 334). Thunberg noted that the stems were terete, striated, hairless, simple, hollow, a foot tall and sub-umbellate at the apex. After the description, he gave a brief diagnosis, segregating his new lily from the *L. canadense* Flor. Japon. P. 135, which he had earlier referred to the Japanese species, i.e. L. leichtlinii subsp. maximowiczii (Thunberg 1784: 135):

“Differt a Lilio canadense: foliis basi latis et corollis minus revolutis.” [differs from *Lilium canadense* in the broader basal leaves and the corollas, which are less revolute].

Also in Thunberg’s Herbarium in Uppsala is a sheet labelled by him “*L. maculatum*” [UPS-THUNB. 8141] comprising an inflorescence of two large, upright campanulate flowers with spotting clearly visible on the tepals. This sheet has two inscriptions written on the recto in Thunberg’s hand: “Lilium martagon” on the bottom left and “Lilium maculatum” on the bottom right. On the verso, there is simply “e Japonia, Thunberg” also in his hand. This sheet is without doubt original material for the name *L. maculatum* Thunb.

Thunberg had included in his *Flora Japonica* what he had believed was the North American *L. philadelphicum* (Thunberg 1784: 135). A decade later, in his Observations, he concluded that his concept of that species was in fact based on *L. bulbiferum* with the description “foliis sparsis lanceolatis, corollis campanulatis erectis, caule villoso” [with sparsely arranged lanceolate leaves, upright campanulate corollas and villose stems] (Thunberg 1794: 333). This description is a slightly modified version of Linnaeus’s protologue for *L. bulbiferum* with the addition of “lanceolatis” for the leaves and “villoso” for the stem (Linnaeus 1753: 302). Again, in the Observations, Thunberg goes on to distinguish what he considered to be the differences between his second identification of *L. bulbiferum* in Japan and his earlier concept of *L. philadelphicum* (Thunberg 1794: 334):

“Differt a lilio philadelphico: 1. foliis basi laterioribus

2. corollis non revolutis”

[differs from *Lilium philadelphicum*: (1). Basal leaves much broader (2). Corollas not revolute].

In Thunberg’s Herbarium in Uppsala, there is another sheet [UPS-THUNB. 8129] consisting of two plants, both with lanceolate leaves and each with a single upright flower, neither of which appears to have spots on the tepals. Each plant has been carefully cut out from its original mount and remounted onto the one sheet. He clearly recognised this as a variety of what he considered to be *L. bulbiferum*. This sheet has two inscriptions written on the recto “bulbiferum β fl. Jap” and ‘Lilium bulbiferum d”. On the verso is written “e Japonia Thunberg. Diff a Philad. foliis basi laterioribus, corollis erectis campanulatis”. Thunberg was therefore distinguishing these from his earlier concept of *L. philadelphicum* as a Japanese variant of the European *L. bulbiferum*L.

In summary, in the Observations, Thunberg had changed his mind about the identification of some of the Japanese lilies that he had included in Flora Japonica (Table 1). His earlier identification of *L. bulbiferum*, he now distinguished as his new species *L. lancifolium*, while recognising a new *L. bulbiferum*, which he then distinguished from his earlier *L. philadelphicum* (see also below under *L. elegans*). Thunberg described his new species, *L. maculatum*, distinguishing it from his earlier concept of *L. canadense*, which is now referable to L. leichtlinii subsp. maximowiczii.

## ﻿Thunberg’s third concept of *Lilium* in Examen Liliorum Japonicorum

Seventeen years after the Observations, Thunberg continued his revision of the Japanese lilies (Table 1) in his *Examen Liliorum Japonicorum* [Examination of the Japanese lilies] in which he included the following eight names: *L. “pomponicum*”, *L. lancifolium*, *L. elegans* Thunb., *L. longiflorum*, *L. maculatum*, *L. japonicum*, *L. speciosum* and *L. cordifolium* (Thunberg 1811: 200–208). His only newly described species was *L. elegans* (Thunberg 1811: 203). In this reappraisal of the eight lilies which he had found in Japan, he clearly stated (translated from Latin):

“After repeated and more accurate examinations and comparing them with other more common species, I easily perceived that there were several dissimilar species that were very different from *L. candidum*, *L. bulbiferum*, *L. canadense* and *L. philadelphicum*. On closer inspection and examination, I found new and unknown species present which I was too timid to describe, daily correcting my own errors, I have endeavoured to correct my mistakes and so began a new survey of Japanese lilies illustrating them with icons for the scrutiny of the botanists of the Imperial Academy of Sciences of St. Petersburg” (Thunberg 1811: 201).

Thunberg does not indicate who it was in the Imperial Academy of Sciences that he expected his descriptions and illustrations of Japanese lilies to be scrutinised by, but he had connections with his mentor Linnaeus’s correspondents in St. Petersburg. Thunberg exchanged plants and insects with the Prussian naturalist Peter Simon Pallas (1741–1811), who had been professor of natural history at the St. Petersburg Academy from 1767. Pallas was clearly much respected by Tsarina Catherine II, who presented to him in 1796, the year of her death, a large estate at Shulyu [Shuli or Ternovka], Simferopol District in Crimea, where he lived until 1810, a year before his own death.

Thunberg corresponded regularly with the Swiss mathematician Nikolaus [Nikolai] Fuss (1725–1826), who had joined the Imperial Academy of Sciences in 1773 and, from 1800 until his death, was the Academy’s permanent secretary (Skuncke 2014: 247). In 1801, Thunberg was elected a foreign member of the Academy (Andrey Sytin pers. comm.). As a foreign member, Thunberg had arranged to have his reappraisal of Japanese lily species figured in the Academy’s Memoirs. A letter to Thunberg conserved in the University of Uppsala Library from Nikolaus Fuss dated 6 May 1810 stated

“*L’Académie a reçu avec beaucoup de reconnaissance le mémoire que vous m’avez transmis sur les lys du Japon. Elle m’a chargé de vous en faire ses remerciements; et pour vous donner une marque de sa gratitude et de son estime*” [The Academy has received with much gratitude the memoir that you sent to me on the lilies of Japan. They have asked me to thank you for it, and to give you a mark of their gratitude and esteem] (Uppsala universitetsbibliotek shelf mark G 300k).

The illustrations accompanying Thunberg’s text include the note “gravé chez Klauber”, bottom right. This refers to Ignaz Sebastian Klauber (1753–1817), who having been court engraver for Clemens Wenceslaus of Saxony, the Archbishop-Elector of Trier, was invited by Count Aleksei Musin-Pushkin president of the Russian Imperial Academy of Arts to teach engraving in St. Petersburg in 1796 and who was the first curator of prints at the Hermitage from 1805 for Tsar Alexander I. Klauber maintained this curatorial role until his death in 1817. On the left of each illustration is written “dessin par Olin”, a reference to Thunberg’s student at the University of Uppsala, Johan Henric Olin (1769–1824), whose dissertation in the Faculty of Medicine was chaired by Thunberg in 1797 and who was known for his production of illustrations for published botanical works (Tchernaja 1994: 356). He was one of the major contributors to the Icones Plantarum Japonicarum, comprising 305 drawings from plants in Thunberg’s herbarium, of which 50 were engraved and published in instalments of ten engravings each in Uppsala between 1794 and 1805. The full set of 305 drawings for the Icones was acquired by Carl Johan Maximowicz (1827–1891) in 1871, two years after his appointment as director of the Academy’s Museum, and is now housed in the Komarov Botanical Institute in St. Petersburg (Skuncke 2014: 288; Sanz Guillén 2023: 841, 842). About half of the illustrations for the Icones were drawn from Thunberg’s herbarium specimens by Olin (Ohba 1994: 408). Neither *L. maculatum* nor *L. elegans* was included in the full set of the *Icones*. In fact, the only Japanese lily that was illustrated was *L. speciosum* by Herstedt.

Thunberg did not send his text on the Japanese lilies to Fuss until several years after 1805, i.e. until after the drawings for the Icones had ceased to be published. Consequently, he will have asked Olin to provide new illustrations of these lilies based on his herbarium specimens of *Lilium* from Japan for the article in the Academy’s Mémoires. Olin’s illustrations were sent from Uppsala to Nikolai Fuss in St Petersburg, who gave them to Klauber, who engraved them for the Mémoires.

Thunberg’s only new species in the Examen was *L. elegans* (Table 1), for which he cited both *L. philadelphicum* from Flora Japonica and “*L. bulbiferum*. Act. Societ. Linnean Londinensis 2. p. 333”, from his Obervations. He also cited “Willdenow Spec. Plantar. 2 p. 85 et 86” as a synonym (Thunberg 1811: 203). Carl Ludwig Willdenow (1765–1812), in fact, stated, “Planta Japonica ab illust. Thunbergio descripta in Actis Soc. Lin. Lond. l.c. videtur a nostra diversa. W.” [The Japanese plant described by the celebrated Thunberg in Act. Soc. Lin. Lond. seems different from ours] (Willdenow 1799: 86). The “ours” being a reference to the European *L. bulbiferum*L. Willdenow also included a description of Thunberg’s *L. maculatum* stating that it differed from *L. canadense* by its broader basal leaves and less revolute corolla (Willdenow 1799: 89).

Thunberg’s brief description for his new *Lilium elegans* was: “foliis alternis oblongis, corolla campanulata, petalis oblongis” adding “*flos* terminalis, solitarius, magnus” and “*corolla* campanulata, incarnata”. He added a diagnosis distinguishing *L. elegans* from the *L. bulbiferum* that he had already described in his Observations:

“*Differt* a Lilio *bulbifero*, cui proximum:

1. *Caule* simplici, laevi et unifloro, nec striato, diviso.

2. *Foliis* magis ovato-oblongis, alternis, remotis.

3. *Petalis* ovatis, nec inferne angustato-unguiculatis. (Thunberg 1811: 203).

Thunberg states that it differs from the bulbiferous lily, which it resembles by having: (1). A simple stem, smooth and single-flowered, not streaked and divided (2). Leaves larger, alternate, ovate-oblong, separated from each other (3). Petals ovate, not narrowly clawed."

**Figure 1.**
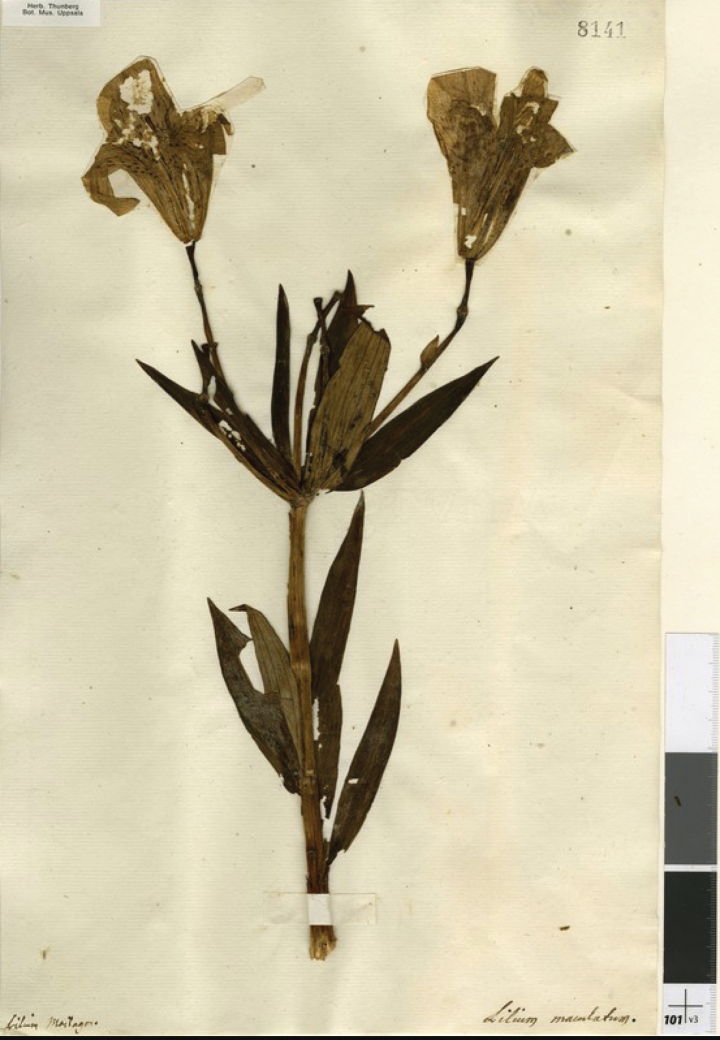
Lectotype specimen of *L. maculatum* [UPS.THUNB.008141].

**Figure 2.**
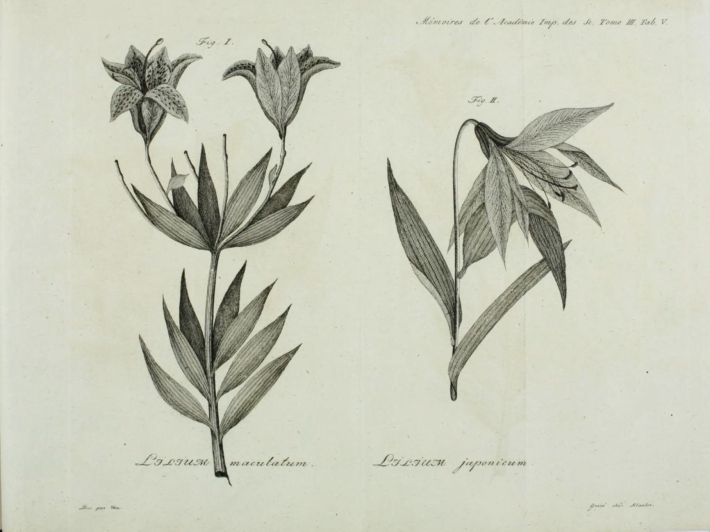
Ignaz Klauber’s engravings in Memoirs of the Academy of St. Petersburg, vol. 3 (1811), of Johan Olin’s illustrations for *L. maculatum* and *L. japonicum* based on Thunberg’s collections in UPS.

The accompanying illustration of *L. elegans* (Fig. 4) is of a single upright, bowl-flowered lily with unspotted tepals and scattered leaves (Thunberg 1811: t. 3 fig. 2). It clearly resembles *L. maculatum*. This was Thunberg’s renaming of his Japanese var. β of *L. bulbiferum*L. Indeed, both of Thunberg’s two specimens in Uppsala comprise UPS-THUNB. 8129 (already discussed above), albeit both with their floral segments partially destroyed and both having been remounted onto a single sheet, are undoubtedly original material for the name *L. elegans*. The left hand of the two specimens was clearly the specimen that provided the exemplar for Olin’s illustration (Thunberg 1811: t. 3 fig. 2). As the artist Olin and consequently, the engraver Klauber were basing their illustrations on Thunberg’s herbarium specimens, the illustration of *Lilium maculatum* (Fig. 2) also published in the Examen and based on the specimen UPS-THUNB. 8141, clearly shows the same overall composition, including the spotting on the tepals (Thunberg 1811: t. 5 fig. 1).

Thunberg provided a more concise description of *L. maculatum* in the *Examen*: “Foliis verticillatis lanceolatis, floribus erectis, corollis campanulatis apice reflexis” [Leaves verticillate, lanceolate, flowers upright, corollas campanulate, apices reflexed] and with added diagnostic comments including “Corollae campanulatae, sanguineae, intus purpureo-maculatae, laciniae apice reflexae” [Corolla campanulate, blood-red, inside purple-spotted, divisions reflexed at the apex] (Thunberg 1811: 204). He still maintained the Japanese names “Kentan”, “Oni Juri” and “Jamma Juri” for this species.

Further evidence is presented of the hand of Olin’s artwork and subsequent engraving by Klauber in the Examen (Thunberg 1811). Their work is clearly evident in the preparations for other illustrations of Thunberg’s *Lilium* descriptions. The illustration of the tiger lily, *L. lancifolium* Thunb. (Fig. 4) was based on the herbarium specimen UPS-THUNB. 8139 (Thunberg 1811: t. 3 fig. 1). In Gao and Gao’s typification of that species, no mention was made by them that the specimen was the exemplar for the illustration (Gao and Gao 2017: 172–174).

## ﻿Summary of Thunberg’s Japanese *Lilium* descriptions

In his original description of *L. maculatum*, Thunberg stated “foliis sparsis et verticillatis” and “*corolla* campanulata limbo reflexo, incarnata intus maculis purpureis plurimis adspersa”, using the term ‘incarnata’, i.e. corolla flesh-coloured or pale pink for the base colour, adding “distributed inside with many purple spots” (Thunberg 1794: 334). The illustration accompanying the protologue of *L. elegans* is merely a black and white line drawing; however, Thunberg’s description for *L. elegans* once again includes the Latin phrase “corolla incarnata” or corolla flesh pink (Thunberg 1811: 203). In the Examen, Thunberg describes *L. maculatum* as having “Corollae campanulatae, sanguineae, intus purpureo-maculatae”, i.e. with blood-red corollas, spotted inside with purple.

**Figure 3.**
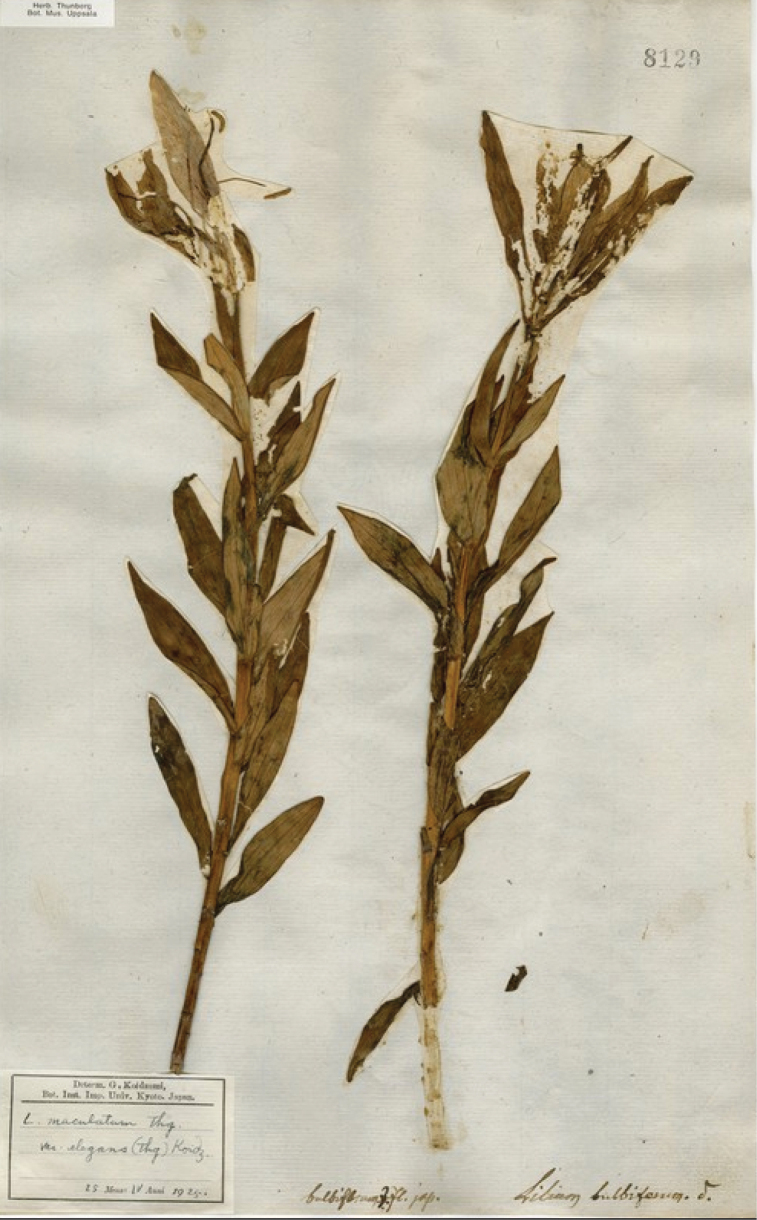
Lectotype specimen of *L. elegans* [UPS.THUNB.008129].

On examination of Thunberg’s two specimens in UPS, it is evident that he was describing two very similar lilies: *L. maculatum* and *L. elegans*. Moreover, the information he supplied in his two protologues and in his 1811 commentary also indicated that he was renaming each lily – *L. maculatum* for *L. canadense* and *L. elegans* for *L. bulbiferum*. He was also renaming *L. elegans* for his earlier determination of it as *L. philadelphicum*. The two Thunberg collections in UPS both represent original material; UPS-THUNB. 8141 (Fig. 1) for the name *L. maculatum* and UPS-THUNB. 8129 (Fig. 3) for the name *L. elegans*. The question is: - Are there any differences between these two taxa? Thunberg’s descriptions of each and the distinctions he made to segregate them from his earlier identifications leave some room for speculation. He mentioned that *L. elegans*, when compared to *L. bulbiferum* has smoother stems and is single-flowered. He stated that *L. maculatum* and *L. elegans* both have pink flowers, although he added later that those of *L. maculatum* can be blood red. He added that the tepals of the latter are spotted, but he does not mention the occurrence of any spotting on the tepals of *L. elegans*. He refers to the leaves of *L. maculatum* as being both sparse and whorled, whereas those of *L. elegans* are oblong and alternate, i.e. sparse.

**Figure 4.**
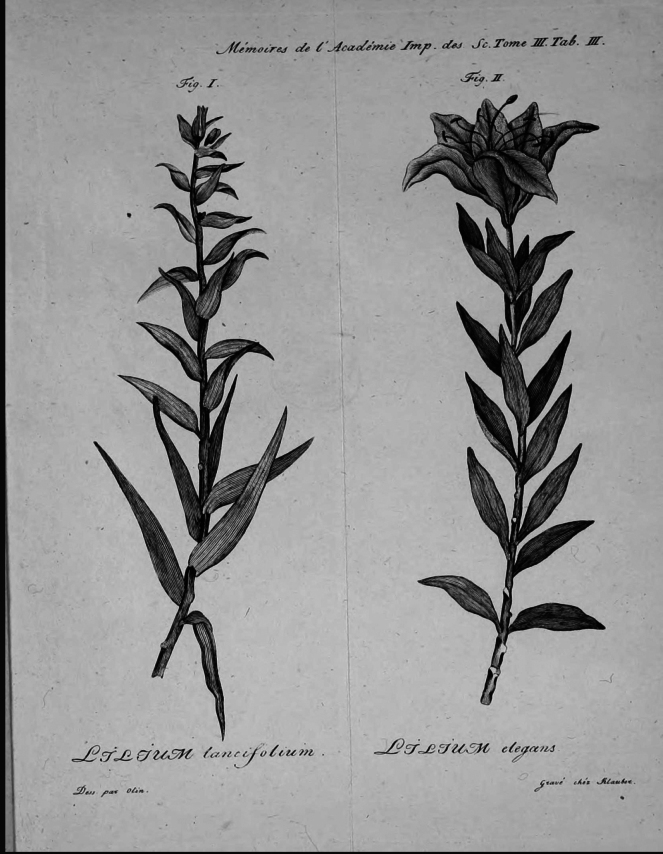
Ignaz Klauber’s engravings in Memoirs of the Academy of St. Petersburg, vol. 3 (1811), of Johan Olin’s illustrations for *L. lancifolium* and *L. elegans* based on Thunberg’s collections in UPS.

Examination of UPS-THUNB. 8141 (Fig. 1) shows that the leaves of this specimen, i.e. *L. maculatum*, are whorled at the junction of the inflorescence and the stem, whereas those on UPS-THUNB. 8129, i.e. *L. elegans* (Fig. 3), are sparsely arranged throughout. When more than a single flower is borne on an inflorescence, as is typical on those of *L. maculatum* and its close relatives *L. bulbiferum* and *L. pensylvanicum*, a whorl of leaves is usually formed at the junction of the peduncle at the point where the pedicels arise. In effect, this whorled region sits at the junction from where the leaves, pedicels and leaf-like bracts all emanate. In the specimen UPS-THUNB. 8141, the inflorescence comprises two flowers on long pedicels and has two additional pedicels that have lost their flowers, with the resulting whorl of six leaves emerging from the base of the four pedicels. The absence of this whorl is inevitably evident on single-flowered plants, such as those on the two specimens on the sheet of UPS-THUNB. 8129. Flower colour on plants in the wild of *L. maculatum* is extremely variable, ranging from orange to red and, in rare cases, yellow. Spotting on the tepals is another variable character that is either present or, less frequently, absent, and the species exhibits variable amounts of pubescence, especially along the floral buds. Examination of the leaves of UPS-THUNB. 8129, i.e. of *L. elegans*, however, shows the presence of papillae along the adaxial midrib and a smooth stem, implying two morphological characters that indicate potential hybridity between *L. pensylvanicum* (smooth stems) and *L. maculatum* (papillose leaf midribs). In conclusion, therefore, it is considered that the latter represents the hybrid taxon L. × elegans (see further discussion below).

### ﻿*Lilium thunbergianum* Schult. & Schult.f.

Joseph August Schultes (1773–1831) and his son Julius Hermann Schultes (1804–1840) commemorated Thunberg by naming a lily after him (Schultes and Schultes f. 1829: 415). Their protologue involved two references to Thunberg’s earlier species names. The first refers to his initial concept of *L. bulbiferum*, which Thunberg later established to be his new species *L. lancifolium* (Thunberg 1794: 333). The Schultes’s included as their second synonym Thunberg’s concept of *L. philadelphicum* i.e. to his second concept of *L. bulbiferum*, which he later described as *L. elegans* (Thunberg 1811: 203).

The Schultes’s third synonym referred to “variety D” of Pierre-Joseph Redouté’s *L. bulbiferum* (Redouté 1808: t. 210). The text for t. 210 in volume 4 of Redouté’s *Les Liliacées* prepared by Augustin Pyramus de Candolle, refers principally to Redouté s plate of the European *L. bulbiferum*L., but the variety D refers directly to Thunberg’s second concept of *L. bulbiferum* in Japan, i.e. to what he later described as *L. elegans*. However, both Candolle and the Schultes’s placed a question mark next to what they described as “L. bulbiferum D, caule non bulbifero? foliis basi latioribus, superioribus verticillatis, inferioribus sparsis”, indicating their uncertainty as to the precise identity of this variety (Redouté 1808: t. 210; Schultes and Schultes f. 1829: 415). In a discussion below the synonymy of *L. thunbergianum* the Schultes’s referred to Willdenow (1799), which was a tangential argument concerning the confusion engendered by Ker Gawler (1805; 1809a; 1809b) regarding what is now *L. pensylvanicum* (Compton and Sytin 2023). The placement of *L. thunbergianum* within the synonymy of *L. elegans* by Watanabe et al. (2024: 469) based on the same type as that of *L. elegans* is entirely reasonable and refers it to the hybrid (see below).

### ﻿Lilium × maculatum

Research undertaken using nuclear DNA ITS sequence data aimed specifically at the question of whether *L. maculatum* is of hybrid origin and what the parents could potentially be, showed that there were 13 polymorphic sites between *L. concolor*, *L. maculatum* and *L. pensylvanicum* (Dubouzet and Shinoda 1999b: 214). Six of these sites were shared between *L. maculatum* and *L. pensylvanicum*, while the remaining seven found in *L. maculatum* could not be attributed to either *L. concolor* or *L. pensylvanicum* (Dubouzet & Shinoda 1999b: 214). *Lilium concolor* and *L. pensylvanicum* shared six of the 13 sites, whereas *L. concolor* and *L. maculatum* differed in all 13 polymorphic sites. In conclusion, there was sufficient molecular divergence between *L. maculatum* and the closely related *L. pensylvanicum* to maintain *L. maculatum* as a separate species (Dubouzet and Shinoda 1999b: 217). They did, however, ask the highly pertinent question – “just how many nucleotide differences are required to confirm the distinctions between section, species and subspecies in *Lilium*?” (Dubouzet and Shinoda 1999b: 217). They implied that the number of base differences is directly proportional to the level of genetic divergence and that, logically, infraspecific variation could be categorised by such taxa having fewer than seven polymorphisms (Dubouzet and Shinoda 1999b: 217). They stated that it was quite possible that populations of *L. maculatum* were the remnants on Honshu of a southerly spread from Hokkaido of *L. pensylvanicum*, and that those continued to speciate after the last glacial period, i.e. c. 10,000 ybp. They posited that these populations developed mechanisms such as immediate epigeal germination that allowed them to compete and survive in the milder, more southerly climate of Honshu (Dubouzet and Shinoda 1999b: 217).

Molecular phylogenies including these three species based on both nuclear and plastid DNA, have shown that *L. maculatum* belongs either on a clade with *L. bulbiferum* and *L. pensylvanicum* (sampled as *L. dauricum*) sister to a clade with *L. concolor* (Nishikawa et al. 2001: 43) or on a clade with *L. lancifolium* and *L. pensylvanicum* sister to a clade with *L. callosum*, *L. concolor* and *L. leichtlinii* (Nishikawa et al. 1999: 248; Dubouzet and Shinoda 1999a: 957; Dubouzet and Shinoda 1999b: 217; Givnish et al. 2020: 39). Additional studies have shown that *L. pensylvanicum* (as *L. dauricum*) sits on a branch with *L. leichtlinii* sister to *L. lancifolium* and *L. maculatum* (Du et al. 2014: 254) or that *L. maculatum* and L. maculatum var. bukosanense formed a branch sister to *L. davidii* with weak bootstrap support (68% maximum parsimony bootstrap and 62% maximum likelihood bootstrap) while *L. pensylvanicum* (as *L. dauricum*) formed a very strongly supported branch with L. lancifolium var. flaviflorum Makino (98% MP and 98% ML), sister to *L. leichtlinii* Hook.f. (Watanabe et al. 2021: 192). In none of these phylogenies is it evident that *L. maculatum* and *L. concolor* occur on the same branch of the relevant clade, nor is there any evidence from morphology that *L. maculatum* exhibits any characters linking the two species.

All the taxa mentioned here belong to an amended Lilium sect. Sinomartagon H.F.Comber (Comber 1949: 101), when it is combined with the monotypic L. sect. Daurolirion H.F.Comber, whose sectional name was based solely on *L. pensylvanicum* (as *L. dauricum*) (Comber 1949: 102). Dubouzet and Shinoda (1999b: 217) concluded that *L. maculatum* and *L. pensylvanicum* are closely related but discrete species, that *L. concolor* is sister to them both, and that *L. maculatum* is not of hybrid origin.

### ﻿Lilium × elegans

Thunberg’s *Lilium elegans* has been suggested to be a hybrid between *L. pensylvanicum* and *L. maculatum* (Shimizu 1960: 105; 1971: 277; 1987: 75; Furuya 2009: 85). The very large range of highly ornamental cultivated material found in Japan bred over many centuries has been the basis of this suggestion. Karyological research cited by Shimizu (1960: 115) indicated that the chromosomes of *L. maculatum* differed from those of *L. pensylvanicum* (as *L. dauricum*). The latter were shown to have secondary constrictions in the seventh pair of chromosomes, whereas those of *L. maculatum* lacked any secondary constrictions (Kumazawa and Kimura 1946). A more recent karyological study on 32 species of *Lilium* in China found that the variation in chromosome morphology across the genus in China as a whole is relatively low (Gao et al. 2011: 749). *Lilium maculatum*, however, was not sampled as that study was restricted solely to Chinese taxa. However, the sample of *L. pensylvanicum* (as *L. dauricum*) included by Gao et al. (2011) did not reveal the presence of secondary constrictions in the seventh pair of chromosomes (Gao et al. 2011: 758). Shimizu, when discussing the morphology of the putative parent species of L. × elegans, i.e. *L. pensylvanicum* and *L. maculatum*, went on to distinguish *L. maculatum* morphologically by its production of underground stolons which can reach more than 10 cm in length before emerging from the soil (Shimizu 1960: 111). This feature, however, was used by Hayashi to refer to *L. pensylvanicum* and to distinguish it from *L. maculatum* in his treatment of *Lilium* in the Flora of Japan (Hayashi 2016: 112). It is likely, however, that both species exhibit underground stolon formation when the conditions for stolon production are favourable, i.e. when plants are growing in fertile, free-draining, loamy soil.

From the information provided by Thunberg (1811: 203) and examination of his herbarium material of *L. elegans*, UPS-THUNB. 8129 (Fig. 3), it is evident that he was describing a plant that varied from *L. maculatum*. There is no evidence as to exactly where Thunberg may have acquired this material, but as it had been long-cultivated in Japan, the assumption must be that it was of garden origin. The question of the attribution of the correct name of any hybrid between *L. pensylvanicum* and *L. maculatum* would therefore be considered here. It is well recognised in Japanese literature that plants have been bred from these species for hundreds of years. In western literature, it was regularly thought that *L. thunbergianum* was the correct name to be used for this hybrid; however, *L. elegans* was described in 1811 and *L. thunbergianum* in 1829 and included the earlier name in synonymy. The name *L. thunbergianum* is therefore considered to be an illegitimate name (Art. 52.1 of the ICN) automatically typified (Art. 7.5 of the ICN) by the type of the name *L. elegans*.

The evidence of hybridisation between *L. maculatum* and *L. pensylvanicum* is extremely well presented based on evidence from sequence data from five plastid genes and two nuclear genes (Watanabe et al. 2024: 458, 459). However, they did not cite a valid hybrid name, Art. H3.1 states that hybrids between two or more taxa may receive a name, and H10.1 states that names of nothotaxa (hybrids) must conform to the provisions of the Code. Art 10.1 Note 1 states that taxa previously published as species may be indicated as hybrid taxa without a change of rank. The first available name for a hybrid between the widespread *L. pensylvanicum* and the more restricted Japanese *L. maculatum* is L. × elegans Thunb. This name can be applied to all horticultural cultivars of hybrid origin with this parentage.

## ﻿Natural variation within *L. maculatum*

Watanabe et al. (2024) recognise two species and four infraspecific taxa within their concept of *L. maculatum* as well as the hybrid between *L. maculatum* and *L. pensylvanicum*. They regard L. maculatum var. maculatum as occurring along the east Tōhoku coast on the north-eastern part of Honshu and on the Sado-Tobishima islands off the west coast of Honshu (Watanabe et al. 2024: 463, 464). They added that *L. maculatum* occurs in coastal areas of Niigata Prefecture of the Chūbu district facing the Japan Sea (Watanabe et al. 2024: 465). These places lie along the northern and western parts of Honshu and would have been far from the areas where Thunberg was permitted to travel in order to collect his lilies during the hofreis to Edo in the summer of 1776. Indeed, his route lay along the Pacific coast of southern Honshu (Kijewski 2015: 162). In effect, Thunberg simply could not have seen *L. maculatum* from those northern and western regions of Japan. Watanabe et al. claimed that *L. maculatum*, which they say is sometimes called in Japanese, “Iwa-yuri” [rock lily], occurs on the coast of West Tōhoku in northern Honshu and includes what they considered to be the type of *L. maculatum* (Watanabe et al. 2024: 465). Moreover, they also claimed that *L. maculatum* had originated genetically by introgression as part of their new species *L. pacificum* S.T.Watan., Fuse & M.N.Tamura, whose holotype came from the Izu peninsula in Shizuoka Prefecture along the Pacific coast (Watanabe et al. 2024: 465). Although there is no evidence that Thunberg visited the peninsula, it is close to his route to Edo along the Pacific east coast.

**Figure 5.**
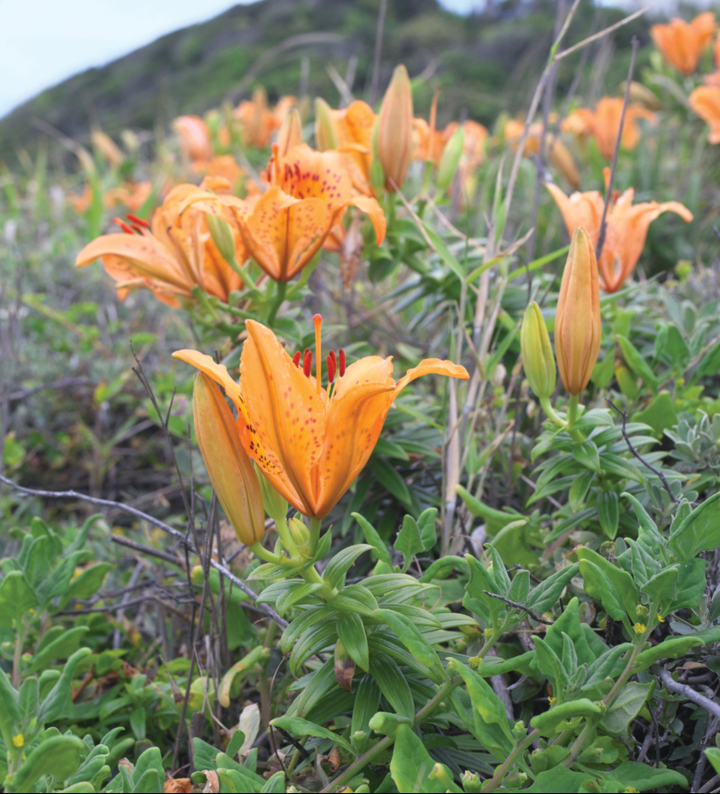
*Lilium maculatum* growing near the east coast along Thunberg’s route past Omaezaki, Shizuoka Pref. Photo. Skycat. 2 June 2021.

Watanabe et al. (2024: 465) distinguished what they understood to be *L. maculatum* from their *L. pacificum* by the presence of papillose hairs on the midribs of the adaxial leaf surfaces of *L. maculatum* (vs. smooth on *L. pacificum*). They described *L. pacificum* from the east coast of Shizuoka Prefecture, and as occurring in coastal parts of Kanto and eastern Chūbu districts of Honshu. These are certainly the regions that Thunberg had passed through on his hofreis journey both to and from Edo (Kijewski 2015). Thunberg’s original material of *L. maculatum* in UPS [UPS-THUNB 8141] presents no evidence of papillose midribs along the midveins on the adaxial leaf surfaces. The material described by Watanabe et al. as *L. pacificum* (Watanabe et al. 2024: 466), lacking papillae and occurring from one of the regions traversed by Thunberg and matching Thunberg’s own original material, therefore represents *L. maculatum* (Fig. 5).

Considering comments concerning the putative recent speciation of *L. maculatum* from a relict population of *L. pensylvanicum* extending south into northern Honshu (Dubouzet and Shinoda 1999b: 217) and the comments by Watanabe et al. (2024: 465) concerning the high degree of gene flow by introgression within *L. maculatum*, it seems sensible to recognise the taxon they understood as *L. maculatum* from coastal regions near the Japan Sea on the west side of Honshu as a distinct taxon. In addition, the levels of variation within *L. maculatum* across the range of the species, as shown by Watanabe et al. (2024: 465), merit recognition of the material described by them (as *L. maculatum*) at the rank of variety. Watanabe et al. (2024: 466) have already described the material from Sado Island and Niigata Prefecture on the west coast of Japan as L. maculatum var. sadoense S.T.Watan., Fuse & M.N.Tamura; consequently, this name is taken up here for the variant with leaves possessing papillae on adaxial (and abaxial) leaf surfaces.

### ﻿Identification key to distinguish *L. concolor*, *L.* × *elegans, L. maculatum* and *L. pensylvanicum*

**Table:** 

1	Flowers stellate in outline, perigone segments usually red, sometimes spotted, (rarely yellow in L. concolor var. coridion (Siebold and de Vriese) Baker) 2.5–6 × 0.5–1.5 cm	***L. concolor***
–	Flowers bowl-shaped in outline, perigone segments 7–10 × 1.5–2.5 cm, plain or spotted	**2**
2	Plants robust, erect, stem base either papillose or smooth, inflorescence umbellate, flowers (3–)5–20, orange, red, pink or yellow	**L. × elegans**
–	Plants erect or decumbent, inflorescence sub-umbellate, flowers 1–5(–10), orange or red, rarely yellow	**3**
3	Stems glabrous on lower portion, ribbed, floccose with long hairs near apex, densely so in leaf axils, flower buds and at the base of tepal ridges	***L. pensylvanicum***
–	Stems papillose scabrid at base, not ribbed, tepal ridges glabrous	***L. maculatum***

### ﻿Identification key to distinguish the varieties of *L. maculatum*

**Table:** 

1	Stems thin, laxly decumbent, leaves linear, 0.4–1.2 cm wide, papillose on midrib, flowers 1 (–2)	**2**
–	Stems stout, erect, leaves lanceolate 1–2 cm wide	**3**
2	Stigma orange	**L. maculatum var. bukosanense**
–	Stigma reddish-brown	**L. maculatum var. monticola**
3	Adaxial leaf midribs papillose	**L. maculatum var. sadoense**
–	Adaxial leaf midribs glabrous	**L. maculatum var. maculatum**

### ﻿Synonymic conspectus

***Lilium maculatum*** Thunb., Trans. Linn. Soc. London 2: 334 (1794). **Holotype**: Japan, “e Japonia Thunberg, Lilium maculatum”, *C.P. Thunberg s.n*. (holo. UPS -THUNB. 8141!) [V-008141].

= Lilium elegans var. spontaneum Makino, J. Jap. Bot. 8: 40 (1932) ≡ L. maculatum f. spontaneum (Makino) S.T.Watan., Fuse & M.N.Tamura, Taxon 73(2): 465 (2024). **Lectotype** designated by Watanabe et al. 2024: Japan, Iwate Pref., Omoe Peninsula, 11–13 September 1929, *T. Makino* s.n. (lecto. MAK 139176).

= *Lilium pacificum* S.T.Watan., Fuse & M.N.Tamura, Taxon 73(2): 466 (2024). **Holotype**: Japan, Shizuoka Pref. Shimoda-shi, 21 June 1979 (fl.), *S. Kitamura* 193 (KYO holo!) [KYO00029149] isotypes: [KYO00029150; KYO00029151].

**Lilium maculatum var. bukosanense** (Honda) H.Hara, J. Jap. Bot. 38(8): 249 (1963). ≡ *Lilium bukosanense* Honda in Nakai, Iconogr. Pl. Asiae Orient. [Toa Shokubutsu Zusetsu] Tokyo 4(3): 404 t. 128 (1942). **Holotype**: Japan, Honshu, Musasi [Chichibu, Saitama Prefecture], “Musashi no Kune, in monte Bukōsan, *Shimizu Daisuke s.n.* 18 July 1941” (TI holo.!) [TI00083088] Japanese name: Miyama Sukashi Yuri

= Lilium maculatum f. lateritium Nakai, J. Jap. Bot. 18: 164 (1942). **Holotype**: Japan, Tokyo, Aogashima Island, 7 July 1932, *M. Takahashi s.n.* (holo. TI) [TI00083089].

**Lilium maculatum var. monticola** H.Hara, J. Jap. Bot. 38(8): 249 (1963) ≡ L. maculatum f. monticola (H.Hara) S.T.Watan., Fuse & M.N.Tamura, Taxon 73(2): 465 (2024). **Holotype**: Cult. Tokyo Botanic Garden *H.Hara* s.n, 14 June 1962. Japan, Honshu, Yamagata Pref., Atagoyama (TI) [TI00083090]

**Lilium maculatum var. sadoense** S.T.Watan., Fuse & M.N.Tamura, Taxon 73(2): 466 (2024). **Holotype**: Japan, Niigata Pref., Sado Island, Aikawaoura, rocky seaside alt. 10 m., 7 June 1991, *K. Hayashi* 80 (KYO holo!) [KYO00029152] isotypes; [KYO00029153, KYO00029155].

***Lilium*** ×***elegans*** Thunb., Mém. Acad. Imp. Sci. St. Pétersbourg Hist. Acad. 3: 203 (1811) ≡ L. maculatum var. elegans (Thunb.) Koidz., Bot. Mag. (Tokyo) 39: 307 (1925) ≡ *L. thunbergianum* Schult. & Schult. f., Syst. Veg. ed. 15bis 7(1): 415 (1829) [*L. bulbiferum* Thunb. “differt a L. philadelphico. In Japonia” was cited] nom. illeg. (Art. 52.1 of the ICN) ≡ L. bulbiferum subsp. thunbergianum (Schult. & Schult. f.) Baker nom. illeg., Gard. Chron. 1871(2): 1034 (1871) ≡ L. bulbiferum var. thunbergianum (Schult. & Schult. f.) Baker nom. illeg., J. Roy. Hort. Soc. [n.s.] 4(1): 43 (1873). ≡ L. dauricum subsp. thunbergianum (Schult. & Schult. f.) E.H.Wilson nom. illeg., Lilies East Asia: 53 (1925). **Lectotype** designated by Watanabe et al. 2024: 469: “e Japonia, Thunberg, diff. a Philad. foliis basi latioribus, corollis erectis campanulatis”, “bulbiferum β Fl. Jap.”, *C.P.Thunberg* s.n. (lecto.! UPS -THUNB. 8129) [V-008129].

= *Lilium fulgens* C.Morren in [Eds. Pierre Corneille Van Géel & Pierre Joseph Auguste Drapiez] Encyclographie du Règne Végétal pt. 6 suite du genre Lis – *Lilium fulgens* t. 10 (1835) ≡ L. bulbiferum var. fulgens (C.Morren) Baker, J. Roy. Hort. Soc. [n.s.] 4(1): 44 (1873) ≡ L. bulbiferum f. fulgens (C.Morren) A.Wallace, Notes Lilies: 71 (1873) ≡ L. elegans var. fulgens (C.Morren) Baker, J. Linn. Soc. Bot. 14: 240 (1875). Lectotype (designated here): [Icon] Encyclographie du Règne Végétal pt. 6 suite du genre Lis – *Lilium* t. 10 (1835)

= *Lilium venustum* F.Neumann, Allgemeine Deutsche Garten-Zeitung 18(20): 157 (16 May 1840). (see Note under *L. venustum* Kunth below).

= *Lilium venustum* Kunth, Enum. Pl. 4: 265 (1843) ≡ L. bulbiferum f. venustum (Kunth) A.Wallace, Notes Lilies: 71 (1873) ≡ L. thunbergianum var. venustum (Kunth) Maxim. ex Franch & Sav., Enum. Pl. Jap. 2: 69 (1879) ≡ L. elegans var. venustum (Kunth) Elwes, Monogr. *Lilium* 5: t. 19 (1878) ≡ *L. dauricum* [as *davuricum*] var. venustum (Kunth) E.H.Wilson, Lilies E. Asia: 52 (1925).

Note 1: *Lilium venustum* F.Neumann, Allgemeine Deutsche Garten-Zeitung 18(20): 157 (16 May 1840) predates *L. venustum* Kunth, Enum. Pl. 4: 265 (1843). Neumann’s description states: “Eine sehr schöne Lilie, die den Bau der gemeinen Feuerlilie (*L. bulbiferum*) hat, sich aber von ihr unterscheidet: 1. dass die Blumen eine blassgelbe Farbe haben, ohne alle Punkte und Fleke; 2. dass die Blumenblätter mit ihren Rändern an einander gelegt bleiben, 3. dass die Blumen sich sehr lange halten, weshalb sie vom Juli bis im September fortblüht und endlich 4. dass sie keine Bulbillen in den Blattwinkeln trägt, weshalb ihre Vermehrung nur langsam vor sich geht” translated as “A very beautiful lily, having the structure of the gemmed fire lily (*L. bulbiferum*), but differing from it: 1. that the flowers are of a pale-yellow colour, without any spots or speckling; 2. that the petals sit with their edges together, 3. that the flowers last for a very long time, which is why it continues to bloom from July to September and finally 4. that it has no bulbils in the axils of the leaves, which is why it will reproduce only slowly”. *Lilium venustum* Kunth, Enum. Pl. 4: 265 (1843) is described as having flowers: “infundibulari-campanulatis, aurantiaco-miniatis coloribus” i.e. with orange-scarlet flowers. This is clearly then based on a different type and is to be considered an illegitimate name Art. 52.1. The following combinations however, are valid (see synonymy above): L. bulbiferum f. venustum (Kunth) A.Wallace; L. elegans var. venustum (Kunth) Elwes; L. thunbergianum var. venustum (Kunth) Maxim. ex Franch & Sav. and *L. dauricum* [as *davuricum*] var. venustum (Kunth) E.H.Wilson.

= *Lilium sanguineum* Lindl., Edwards’s Bot. Reg. 32 [n.s. 9] t. 50 (1846). ≡ L. thunbergianum f. sanguineum (Lindl.) Baker & Dyer, Gard. Chron. 1872: 1356 (1872) ≡ L. bulbiferum f. sanguineum (Lindl.) A.Wallace, Notes Lilies: 71 (1873) ≡ L. bulbiferum var. sanguineum (Lindl.) Baker, J. Roy. Hort. Soc., [n.s.] 4(1): 43 (1873) ≡ L. elegans var. sanguineum (Lindl.) Baker, J. Linn. Soc. Bot. 14: 239 (1875) ≡ *L. dauricum* [as *davuricum*] f. sanguineum (Lindl.) E.H.Wilson, Lilies E. Asia: 55 (1925). Lectotype designated by Watanabe et al. 2024: 468: [icon] Edwards’s Bot. Reg. 32 [n.s. 9] t. 50 (1846)

= Lilium fulgens var. maculatum Spae, Mém. Couronnés Mém. Savants Etrangers Acad. Roy. Sci. Bruxelles 19(5): 20 (1847). **Type**: Siebold ex Japan s.n. 1825–1830 fl. Ghent Botanic Garden 1833 (not seen).

= Lilium fulgens var. staminosum Lem., Ill. Hort. 11: t. 422 (1864). Lectotype designated here: [Icon] “In horto Verschaffelt, P. Stroobant pinxit” Ill. Hort. 11: t. 422 (1864).

= *Lilium formosum* Lem., Ill. Hort. 12: t. 459 (1865). Lectotype designated here: [icon] “P. Stroobant ad nat. pinxit. In horto Verschaffelt. Japon (plein air)”, Ill. Hort. 12: t. 459 (1865).

= *Lilium haematochroum* Lem., Ill. Hort. 14: t 503 (1867) ≡ L. bulbiferum f. haematochroum (Lem.) A.Wallace, Notes Lilies: 71 (1873). Lectotype designated by Watanabe et al. 2024: 469: [Icon] “In horto Verschaffelt, P. Stroobant pinxit, Japon air libre” Ill. Hort. 14: t 503 (1867)

= Lilium thunbergianum var. pardinum T.Moore, Florist & Pomologist ser. 3(1): 121 (1868) ≡ L. × wilsonii Leichtlin ex R.Hogg, T.Moore & W.Paul, Florist & Pomologist ser. 3(1): 192 (1868) ≡ L. bulbiferum f. wilsonii (Leichtlin ex R.Hogg, T.Moore & W.Paul) A.Wallace, Notes Lilies: 70 (1873) ≡ L. bulbiferum var. wilsonii (Leichtlin ex R.Hogg, T.Moore & W.Paul) Baker, J. Roy. Hort. Soc. [n.s.] 4(1): 43 (1873) ≡ L. elegans var. pardinum (T.Moore) Baker, J. Linn. Soc. Bot. 14: 239 (1875) ≡ *L. dauricum* [as *davuricum*] f. pardinum (T.Moore) E.H.Wilson, Lilies E. Asia: 52 (1925). Lectotype designated by Watanabe et al. 2024: 470: [icon] Florist and Pomologist 1868: 121 t. “Lilium thunbergianum pardinum” (1868).

Note 2: Lilium × wilsonii Leichtlin ex R.Hogg, T.Moore & W.Paul.

As stated in the protologue for *L. thunbergianum* [unranked] *pardinum*, this is of hybrid origin published by Thomas Moore (1821–1887) in the June issue of Florist and Pomologist for the year 1868. The new combination Lilium × wilsonii published in the same volume but in August that same year, is a renaming of the unranked infraspecific name published earlier in June. The rather broad leaves with five longitudinal veins and the dark papillose markings within the basal parts of each tepal, combined with the deep yellow zone along the sinus, suggest the possibility that *L. auratum* Lindl. may have crossed with the upright bowl-shaped reddish-orange flowers of *L. maculatum*.

= *Lilium alternans* Siebold ex Duch., J. Soc. Natl. Hort. France ser. 2 vol. 4 (4): 215, 473 (1870).

= Lilium thunbergianum var. bicolor T.Moore, Floral Mag. 9: t. 504 (1870) ≡ L. bulbiferum f. bicolor (T.Moore) A.Wallace, Notes Lilies: 70 (1873) ≡ L. bulbiferum var. bicolor (T.Moore) Baker, J. Roy. Hort. Soc., [n.s.] 4(1): 43 (1873) ≡ L. elegans var. bicolor (T.Moore) Baker, J. Linn. Soc. Bot. 14: 239 (1875). **Lectotype** designated by Watanabe et al. 2024: 469: [icon] Floral Mag. 9: t. 504 (1870).

= Lilium thunbergianum var. atrosanguineum Baker & Dyer, Gard. Chron. 1872: 1356 (1872) ≡ L. bulbiferum f. atrosanguineum (Baker & Dyer) A.Wallace, Notes Lilies: 71 (1873) ≡ L. bulbiferum var. atrosanguineum (Baker & Dyer) Baker, J. Roy. Hort. Soc., [n.s.] 4(1): 43 (1873) ≡ L. elegans var. atrosanguineum (Baker & Dyer) Baker, J. Linn. Soc. Bot. 14: 240 (1875) ≡ *L. dauricum* [as *davuricum*] f. atrosanguineum (Baker & Dyer) E.H.Wilson, Lilies E. Asia: 55 (1925).

= Lilium thunbergianum f. brevifolium Baker & Dyer, Gard. Chron. 1872: 1356 (1872) ≡ L. bulbiferum var. brevifolium (Baker & Dyer) Baker, J. Roy. Hort. Soc., [n.s.] 4(1): 43 (1873) ≡ L. elegans var. brevifolium (Baker & Dyer) Baker, J. Linn. Soc. Bot. 14: 239 (1875)

= Lilium thunbergianum f. alutaceum Baker & Dyer, Gard. Chron. 1872: 1356 (1872) ≡ L. bulbiferum f. alutaceum (Baker & Dyer) A.Wallace, Notes Lilies: 70 (1873) ≡ L. bulbiferum var. alutaceum (Baker & Dyer) Baker, J. Roy. Hort. Soc., [n.s.] 4(1): 43 (1873) ≡ L. elegans var. alutaceum (Baker & Dyer) Baker, J. Linn. Soc. Bot. 14: 239 (1875) ≡ *L. dauricum* [as *davuricum*] f. nigro-maculatum E.H.Wilson, Lilies E. Asia: 52 (1925) ≡ L. maculatum f. alutaceum (Baker & Dyer) Nakai, J. Jap. Bot. 18: 166 (1942). Lectotype designated by Watanabe et al. 2024: 468: [icon] “L. thunbergianum aureum nigro-maculatum Van Houtte” Fl. Serres 16: t. 1627 (1865).

= Lilium thunbergianum f. armeniacum Baker & Dyer, Gard. Chron. 1872: 1356 (1872) ≡ L. bulbiferum f. armeniacum (Baker & Dyer) A.Wallace, Notes Lilies: 71 (1873) ≡ L. bulbiferum var. armeniacum (Baker & Dyer) Baker, J. Roy. Hort. Soc., [n.s.] 4(1): 43 (1873) ≡ L. elegans var. armeniacum (Baker & Dyer) Baker, J. Linn. Soc. Bot. 14: 239 (1875).

= Lilium thunbergianum f. brevifolium Baker & Dyer, Gard. Chron. 1872: 1356 (1872) ≡ L. bulbiferum f. brevifolium (Baker & Dyer) A.Wallace, Notes Lilies: 70 (1873) ≡ L. bulbiferum var. brevifolium (Baker & Dyer) Baker, J. Roy. Hort. Soc., [n.s.] 4(1): 43 (1873) ≡ *L. dauricum* [as *davuricum*] f. brevifolium (Baker & Dyer) E.H.Wilson, Lilies E. Asia: 55 (1925).

= *Lilium dauricum* [as *davuricum*] var. croceum Regel, Gartenfl. 21: 295 (1872)

= Lilium thunbergianum var. aurantiacum Regel, Gartenfl. 21: 295 (1872)

= Lilium thunbergianum var. stamineum Regel, Gartenfl. 21: 295 (1872) ≡ L. elegans var. stamineum (Regel) Voss, Vilm. Blumengärt. ed. 3 vol. 1: 1099 (1896).

= Lilium thunbergianum var. leichtlinii Regel, Gartenfl. 21: 295 (1872)

= Lilium thunbergianum f. citrinum Baker & Dyer, Gard. Chron. 1872: 1356 (1872) ≡ L. bulbiferum f. citrinum (Baker & Dyer) A.Wallace, Notes Lilies: 71 (1873) ≡ L. bulbiferum var. citrinum (Baker & Dyer) Baker, J. Roy. Hort. Soc., [n.s.] 4(1): 43 (1873) ≡ L. elegans var. citrinum (Baker & Dyer) Baker, J. Linn. Soc. Bot. 14: 239 (1875) ≡ *L. dauricum* [as *davuricum*] f. citrinum (Baker & Dyer) E.H.Wilson, Lilies E. Asia: 55 (1925).

= Lilium elegans var. incomparabile Elwes, Monogr. *Lilium* 5: t. 20 (1878). Lectotype designated by Watanabe et al. 2024: 469: [icon] Elwes, Monogr. *Lilium* 5: t. 20 (1878).

= *Lilium batemanniae* A.Wallace, The Garden 15: 396 (1879) ≡ L. elegans var. batemanniae (A.Wallace) Baker, J. Roy. Hort. Soc. [London] [n.s.] 26: 339 (1901–1902) ≡ *L. dauricum* [as *davuricum*] f. batemanniae (A.Wallace) E.H.Wilson, Lilies E. Asia: 52 (1925). Lectotype: designated by Watanabe et al. 2024: 469 [Icon] The Garden 15: 396, Mrs Bateman’s Lily t. clxxx (1879).

= *Lilium dauricum* [as *davuricum*] f. pluriflorum Voss, Vilm. Blumengärt. ed. 3 vol. 1: 1097 (1896).

= *Lilium wallacei* R.W.Wallace, The Garden 51: 80, t. 1103 (1897) ≡ L. elegans var. wallacei (R.W.Wallace) Waugh, Cycl. Am. Hort. 2(c3): 919 (1900) ≡ *L. dauricum* [as *davuricum*] var. wallacei (R.W.Wallace) E.H.Wilson, Lilies E. Asia: 52 (1925). Lectotype designated by Watanabe et al. 2024: 469: [icon] The Garden 51: 80, t. 1103 (1897).

= *Lilium dauricum* [as *davuricum*] f. aureum E.H.Wilson, Lilies E. Asia: 55 (1925).

= *Lilium dauricum* [as *davuricum*] f. horsmannii E.H.Wilson, Lilies E. Asia: 55 (1925).

- *Lilium aurantiacum* Paxton, Paxton Bot. Mag. 6: 127 (1839) nom. illegit. (*L. aurantiacum* Weston Bot. Univ. 3: 453. 1772 = *L. bulbiferum*L.) Art. 52.1.

- *Lilium atrosanguineum* H.Vilm., Fl. Pleine Terre ed. 3: 629 (1870) nom. nud. in syn.

- Lilium thunbergianum var. aureum J.H.Krelage ex Duch., J. Soc. Natl. Hort. France ser. 2 vol. 4 (3): 353 (1870) nom. nud. without description.

- *Lilium wilsonii* G.F.Wilson ex Baker & Dyer, Gard. Chron. 1872: 1356 (1872) nom. illegit. (Predated by *L. wilsonii* Leichtlin ex R.Hogg, T.Moore & W.Paul, Florist & Pomologist ser. 3(1): 192. 1868) Art. 52.1.

- *Lilium fulgens* Baker & Dyer, Gard. Chron. 1872: 1356 (1872) nom. illegit. (Predated by *L. fulgens* C.Morren, Encyclographie du Règne Végétal pt. 6 suite du genre Lis – *Lilium fulgens* t. 10. 1835) Art. 52.1 ≡ *L. dauricum* [as *davuricum*] f. fulgens (Baker & Dyer) E.H.Wilson, Lilies E. Asia: 54 (1925).

- *Lilium thunbergianum var. aureum nigropunctatum* Rob., Garden 6: 142 (1874) nom. invalid Art. 23.6c.

- Lilium thunbergianum var. splendens Mast., Gard. Chron. N.s. 3: 728 (1875) nom. nud. without description.

- Lilium elegans var. horsmannii E.J.Krelage, J. Roy. Hort. Soc. 26: 356 (1901–1902) nom. nud. without description.

- Lilium dauricum var. furuyanum M.Furuya, Lily Yearb. N. Amer. Lily Soc. 57(4): 6 (2004); Lily Yearb. N. Amer. Lily Soc. 62: 87 (2009) (nom. invalid, no type Art. 7.11, Watanabe et al. 2024: 467 have attributed this name to *L. pensylvanicum*).

- Lilium dauricum var. bihoroanum M.Furuya, Lily Yearb. N. Amer. Lily Soc. 57(4): 7 (2004); Lily Yearb. N. Amer. Lily Soc. 62: 88 (2009) (nom. invalid, no type Art. 7.11, Watanabe et al. 2024: 467 have attributed this name to *L. pensylvanicum*).

## ﻿Additional information

### Use of AI

No use of AI was reported.

### Data availability

All of the data that support the findings of this study are available in the main text.
